# Cost, Draping, Material and Partitioning Optimization of a Composite Rail Vehicle Structure

**DOI:** 10.3390/ma15020449

**Published:** 2022-01-07

**Authors:** Daniel Lang, Donald W. Radford

**Affiliations:** College of Engineering, Colorado State University, Fort Collins, CO 80523, USA; dradford@rams.colostate.edu

**Keywords:** composites, topology optimization, carbon fiber, glass fiber, structural partitioning, draping simulation

## Abstract

This study proposes a novel methodology to combine topology optimization and ply draping simulation to partition composite structures, improve structural performance, select materials, and enable more accurate representations of cost- and weight-efficient manufacturable designs. The proposed methodology is applied to a structure as a case study to verify that the methodology is effective. One design concept is created by subjecting the structure to a kinematic ply draping simulation to inform the partitioning of the structure, improve drapability and performance, and reduce structural defects. A second design concept is created that assumes that plies are draped over the entire structural geometry, forming an integral design. The two design concepts’ topologies are subsequently optimized to specify ideal material and ply geometries to minimize mass and reduce costs. The results indicate that the partitioned structure has a 19% lower mass and 15% lower material costs than the integral design. The two designs produced with the new methodology are also compared against two control designs created to emulate previously published methodologies that have not incorporated ply draping simulations. This demonstrates that neglecting the effects of ply draping produces topology optimization solutions that under-predict the mass of a structure by 26% and costs by 38%.

## 1. Introduction

Fiber reinforced composites possess high specific properties that can offer weight savings over common materials, such as steels, in structural design [1]. These weight savings are particularly attractive to the transportation sector because reducing vehicle weight saves energy [2]. As a result, many vehicle manufacturers have begun replacing metallic materials with fiber reinforced composites. The aircraft industry, for example, has replaced up to 50% of metallic components within an airplane structure with composite materials, which has led to a 21% reduction of fuel consumption [3]. Despite the success achieved in the aircraft industry, major application of fiber reinforced composites in vehicles for other transportation sectors remains limited. For example, even with projections that a train with a structure primarily composed of composites could reduce greenhouse gas emissions by 26% compared to traditional steel designs, there are few rail vehicles that include composite structural components [2].

The primary causes for lack of adoption that have been cited are unfamiliarity with the complexities of composite manufacturing, materials, and their associated costs [4]. This lack of composites-specific experience has become a major impediment to adoption as the projected gains in performance through the implementation of composites revolves around the recognition that the material must be designed in parallel with the structure, and that the form of manufacture affects this optimization. Thus, unlike structures development utilizing more traditional materials, efficient, cost-effective structures developed with composite materials must simultaneously optimize the structural geometry, the material and the manufacturing approach.

Material optimization has impeded composite application because of how different composites are from traditional materials with respect to costs, performance and shape flexibility. Fiber reinforced composites are typically more expensive, on a per mass basis, than traditional structural materials such as steel. Fiber reinforced composites are also available in a variety of types, with large variations in cost and material properties, complicating the design process [5]. Furthermore, composites can be molded into complex structural shapes not possible with metallic materials. Additionally, composites can be easily layered with changing section thickness through ply shape tailoring, which would require expensive and time-consuming machining to accomplish with metals. Industries that are more experienced in composite application have developed processes to incorporate multi-material structures that optimize material usage and structural shapes to balance between the mass savings offered by high performance composites and financial savings possible with lower cost materials [6].

Manufacturing optimization has also impeded application, as processes for composites differ significantly from the metallic material production processes more familiar to the majority of structural designers. In particular, the manufacturing process of ply draping that occurs during lay-up and its effects on structural performance, manufacturability, and costs are not understood by many industries [7]. For example, common practice in industries that have experience with composites is partitioning large, complex, structural geometries into smaller ply areas. This reduces manufacturing defects that could occur if plies were draped over the entire complicated structural shapes. Once partitioned, however, joints between the sections must be carefully designed to provide adequate structural performance. Aircraft designers have developed methodologies to design effective joints between partitioned sections [8]; however, industries that lack experience often neglect these design details [9].

The lack of knowledge of these composite manufacturing and material details can lead to the design of structures that do not achieve the weight savings or cost targets predicted through flawed modeling efforts [10]. The aircraft industry has overcome these issues by developing simulation tools to evaluate composite structures. For example, a research group developed a custom simulation tool for Boeing to assist with composite design [11]. This simulation tool allows Boeing to evaluate major aspects of composite structures such as draping, costs, weight, materials, and partitioning. The simulation tool can also be used to conduct analyses and optimization to develop finalized designs. Similar tools have been developed by Northrop Grumman and other aerospace designers [12]. These tools have assisted aircraft manufacturers in achieving a greater level of composite application in structural design than many other industries through designs that carefully balance the many competing design requirements of composite structures.

While the function of these simulation tools, and the successes achieved by the aircraft industry using them, have been published, they are proprietary to the companies who have developed them [12]. Furthermore, the tools have primarily been developed to assess structures specific to the aircraft industry such as fuselages and wings. Therefore, these established tools are not available or applicable for other industries. The existence of these tools, though, demonstrate a clear demand for their functionality by designers. Designers in other industries have begun researching and developing methodologies that use commercially available software to accomplish similar function to those developed by the aircraft industry [13]. Two commercially available simulation tools specifically that have been the most widely applied in research are topology optimizers and ply draping simulators [14].

Commercially available topology optimization software has been developed to help designers effectively use material to reduce the cost and weight of composite structures. Topology optimization is effective in developing designs that exploit the material properties and geometric flexibility of composite materials [15]. Topology optimization algorithms are embedded in many common Finite Element Analysis (FEA) programs and work through an iterative erosion process reducing initial ply dimensions and modifying section to achieve objectives of reducing material costs and mass of the design. This process mimics the ability to cut and tailor individual plies into complex shapes as part of a laminated composite structure. Optimization algorithms can also be formulated to simultaneously assist in composite joint design and the selection of cost-efficient composite materials, although this has not been explored in research.

Despite topology optimization providing many similar functions to simulation tools developed by the aircraft industry, most research has only explored using topology optimization to fulfill one function at a time [16]. Recently, however, research has begun to integrate multiple functions provided by topology optimization simultaneously to develop designs that are comparable to those produced for the aircraft industry.

To date, the greatest degree of functional integration, was achieved by Mårtensson et al. in a series of published works [17,18]. In these studies, structures were partitioned to reduce the mass and costs of the design. The authors considered flat composite panels to have the least complexity, resulting in the lowest manufacturing costs, while parts with greater depth and feature complexity as more challenging and expensive to produce. The research defined an integral structure as one where, prior to optimization, ply dimensions and the overall structural dimensions were identical. An integral design model of an automotive structural component was created based on this definition. Next, the studies partitioned the same component to create another design model with smaller substructures that had reduced complexity in comparison to an integral approach. The research then conducted topology optimization to find optimal ply sizing within each of the partitioned and integral structures with the goal of reducing mass and costs. Partitioning and topology optimization led to cost savings of over 40% and reductions in mass of 7% when compared to an integral design [18].

Despite the success demonstrated in those studies, further functional integration would be necessary to achieve the same level of design support as that offered by aircraft design tools. This could be achieved through formulating the topology optimization process to support material selection and joint design. Additionally, these studies did not incorporate ply draping simulation to inform the partitioning of the structure and fulfill the final functions offered by aircraft design tools. Ply draping software has been developed to help designers better understand composite manufacturing and to simulate the distortion of the fiber paths that occur during the hand lay-up process [19]. Ply draping analysis has been proven to accurately predict structural defects, such as wrinkling and fabric shear, that may occur during composite manufacturing. Kinematic draping capability is included in many ply draping programs and can assist designers in partitioning structures into subregions with simpler geometries, reducing structural defects [20]. Draping and structural partitioning may also improve weight savings and cost efficiency of composite structures by improving structural performance through reduction of defects [21].

In a more recent study by Mårtensson et al. [21], the benefits of draping simulations in composite design were demonstrated. Kinematic draping was conducted to identify the optimal strategy for partitioning an automotive structure to improve manufacturability and reduce defects. Similar to the topology optimization studies, the partitioned structure was compared to an integral design; it was determined that the partitioned structure offered 10% reduction in costs but a 13% increase in mass compared to the integral design. The increase in mass was due to the added weight of material in the connection regions which was not optimized during the study. Furthermore, this study did not attempt to integrate the process with topology optimization and the previously demonstrated benefits of its functionality which may have resulted in mass savings.

**Figure 1:**
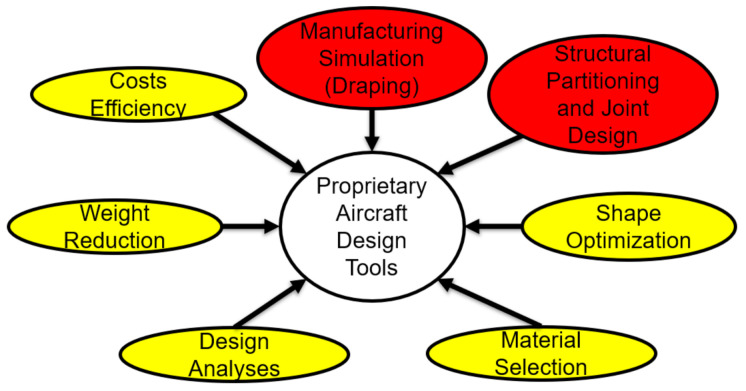
Common design and optimization functions completed by proprietary aircraft design tools. Comparable functionality offered by commercially available topology optimization shown in yellow, and draping simulators shown in red.

Based on the results presented in previous research, integration between topology optimization and ply draping is logical. To date, however, the methodologies developed around the two commercially available simulation software packages have not been integrated to provide comparable functionality to the tools developed by the aircraft industry. Figure 1 illustrates the major design and optimization functions typically completed using proprietary composite aircraft design tools. The functionality provided by draping simulators is shown in yellow in the figure, while the functionality offered by topology optimization is shown in red, demonstrating that comprehensive functionality is possible with further integration of commercially available software.

Applying these processes together may offer designers benefits that lead to improvements in cost and weight efficient designs [18]. This is because topology optimization may further improve drapability of plies and overall structural performance above what can be achieved by performing kinematic draping processes to partition a structure. A methodology that integrates topology optimization with ply draping may lead to increased adoption of composite materials by industries that lack experience with them. Additionally, the integrated methodology may assist in the development of designs that better balance between the many competing design requirements of composite structures, as has been accomplished in the aircraft industry.

This research advances the design of structures made from composite materials in two ways. First, topology optimization methodology is advanced by introducing ply draping analyses as a method of improving the weight savings, manufacturability and affordability of composite structures. Second, a topology optimization function was developed, which aids in joint design and the selection of fiber reinforced composite materials to further reduce the costs and weight of structures. These methodologies are applied to a complex, heavily-loaded rail vehicle structure to demonstrate the value of these advancements over existing optimization processes.

## 2. Materials and Methods

**Figure 2:**
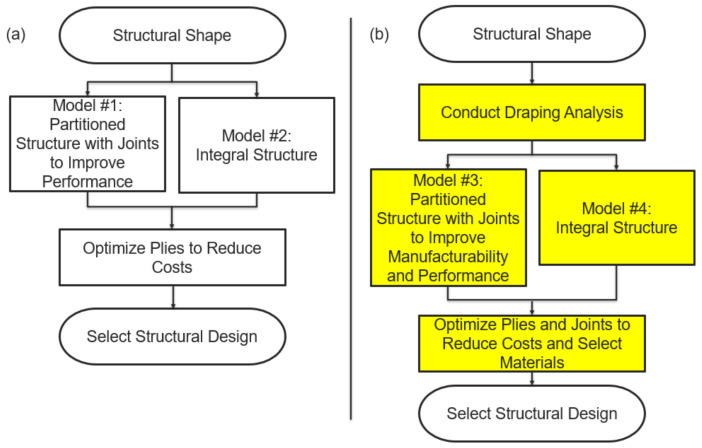
Segmentation and optimization approach applied in previous work (**a**) [18] compared to proposed methodology for this study (**b**).

As explained in the previous section, researchers from industries that lack experience with composites are currently leveraging commercially available software tools in an attempt to achieve levels of composite application comparable to the aircraft industry. The furthest level of integration achieved combined structural partitioning with topology optimization to reduce the costs and weight of a structure [17,18]. These studies, however, have not integrated draping simulations, material selection, or joint optimization. In the current study, the methodology developed in previous work is expanded to demonstrate the importance of combining topology optimization and material selection with draping analyses to develop partitioned designs that have reduced mass and production costs. The general methodologies published as part of previous research [18] are compared to the expanded process of the current study in Figure 2. The general process used in previous research by Mårtensson et al. [18] is used as a control in this study (Figure 2a) to demonstrate the importance of the expanded optimization processes developed as part of the current research (Figure 2b).

Figure 2b highlights in yellow the major advancements made with the proposed methodology compared to previously published works [18]. First, ply draping simulation was used to accurately simulate composite manufacturing and identify any defects that may develop as part of that process. This process has not been incorporated in topology optimization research to date. Next, structural partitioning and modeling of composite joints was conducted to improve both manufacturability and performance. In past studies, partitioning and joint design was completed without respect to manufacturing. Finally, the complete design, including joints, developed with the proposed methodology was optimized to reduce costs and assist in composite materials selection. Past research has not used topology optimization to inform composite joint design between partitioned sections or used the optimization process to inform material selection.

Similar to the studies published by Mårtensson et al. [17,18], this research compares the effects of integral and partitioned structures. As defined in those studies, integral structures are ones where, prior to topology optimization, ply area is identical to the overall structural shape. A partitioned structure, on the other hand, is one where the overall structural shape is divided into smaller ply areas to improve structural performance. The topology optimization studies by Mårtensson et al. [17,18] partitioned structures only to reduce weight and costs. In this study, however, the structures were partitioned through ply draping to provide more realistic representations of manufacturable designs. To compare the effects of these changes, four structural models were developed: one integral and one partitioned model that both neglect the effects of ply draping, representative of the methodology published by Mårtensson et al. [17,18], and one integral and one partitioned structure that both incorporate the results of ply draping, representative of the methodology proposed in this research. The four models compared in this research are shown in Figure 2.

Of importance in the proposed methodology is the organization of the processes. Draping analyses must be conducted prior to topology optimization. This is because topology optimization alters ply geometry in response to design objectives and material performance. Therefore, the plies must be modeled accurately to represent manufacturable performance prior to optimization. With draping conducted first, the topology optimization will be conducted with respect to any structural defects that may occur during ply draping simulation and alter the design accordingly to still meet the design objectives and requirements established. Without draping conducted first, the topology optimization process may develop solutions that overestimate the level of mass and cost reductions that are feasible for the designs. It is expected that control designs generated through the methodologies previously published, shown in Figure 2a, will demonstrate this point, as it excludes ply draping simulations.

It is envisioned that the advancements offered by the proposed methodology in this research will lead to improved balance between manufacturing, materials, costs, mass, and structural performance in the designs developed. Furthermore, it is anticipated that the solutions developed will more accurately represent manufacturable designs based on ply draping simulations. It is further hypothesized that topology optimization can be a supporting process for ply draping and partitioning analysis as it can inform how to tailor ply shapes to improve drapability. It is also hypothesized that a topology optimization algorithm can be formulated to simultaneously assist in material selection, joint design, and improve the cost and weight savings achieved by a structural design. The final hypothesis is that structures optimized under previously published methodologies may overestimate mass and costs savings that are achievable by neglecting the effects of ply draping.

### 2.1. Case Study Structure

**Figure 3:**
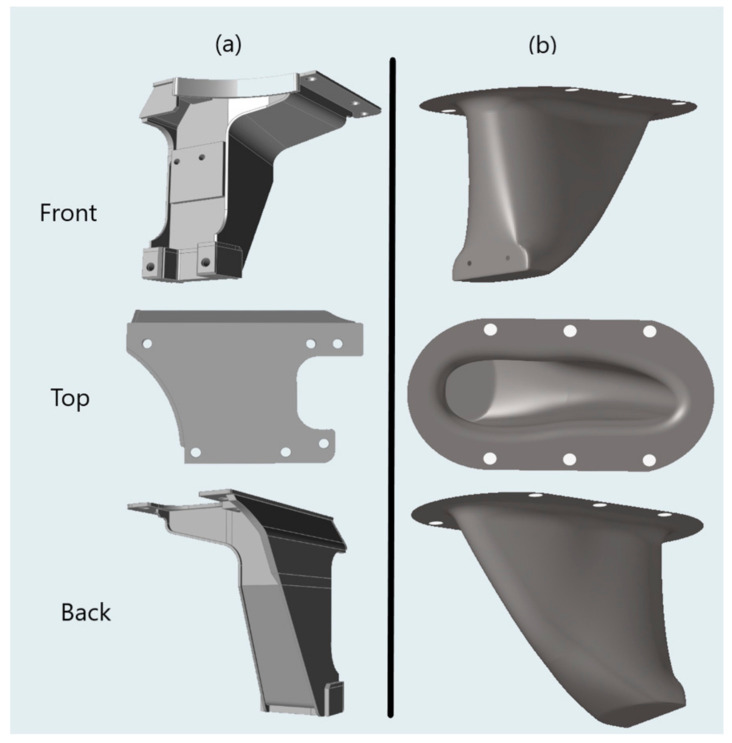
Typical steel anchor bracket (**a**), and composite anchor bracket (**b**) designed to replace the steel design.

To assess the effectiveness of the proposed methodology, a complex rail vehicle structure was selected as a case study. An emerging research trend in many industries, including rail vehicle design, is to investigate replacing steel components with composite structures [22]. However, a direct replacement, using the same geometry as the baseline steel structure is seldom possible using composite manufacturing processes [4]. This research evaluates the potential for replacing a steel rail vehicle anchor bracket with a revised geometry concept that was designed with composite materials in mind. Anchor brackets are common structural components of rail vehicles and are typically manufactured from steel. The function of an anchor bracket is to provide stiffness to efficiently absorb loads developed from propulsion and braking and transmit them out of a rail bogie. These loads are then distributed into the main structure of the rail vehicle. Figure 3 shows a typical steel anchor bracket and the replacement composite structure that is the focus of this case study. While the geometry of the composite concept deviates from that of the baseline steel bracket, the composite component fits within the current rail vehicle design.

**Figure 4:**
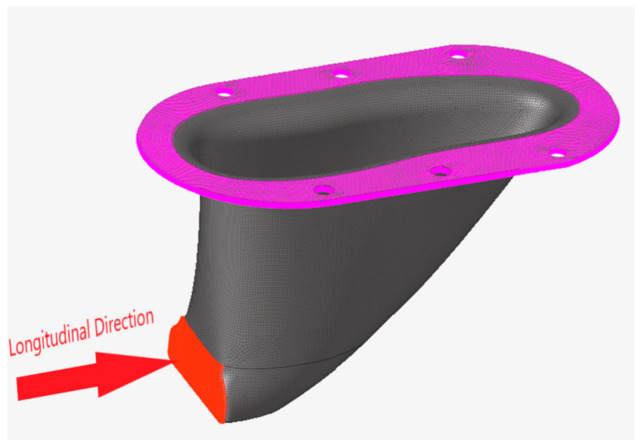
Boundary constraint location at the top of the structure (pink) and load application location on the front, bottom of the structure (red) and the longitudinal load application direction.

Altair HyperWorks FEA software was used to model the composite structure in a manner consistent with current industry practices [23]. The FEA model of the structure, shown in Figure 4 represents the load application and boundary constraint locations applied to the structure. Based on industry standards, anchor brackets are designed to support a static load of 30 kN applied longitudinally [24].

### 2.2. Materials and Manufacturing

The FEA models developed during this study were created assuming common carbon fiber and E-glass fiber reinforced epoxy prepreg materials. The fiber reinforced composite materials were also modeled in two common feedstocks: uni-directional tape and plain weave bi-directional prepreg fabrics. The material properties were obtained from testing reports, and the cost metrics were extracted from a composite manufacturing simulation software program, SEER-MFG from Galorath [25]. The material properties and costs assume that the structure would be manufactured through a hand layup and autoclave process. While other materials and manufacturing processes could have been considered, these were selected to be consistent with other research focused on rail vehicle composite structural design [26].

### 2.3. Material Orientation, Thickness, Ply Draping and Partitioning

Common practice when modeling composite structures is to define fiber orientation and ply thickness. Most published research models composite structures through FEA but neglects the effects of ply draping. Instead, these studies have the fiber directions aligned with respect to a model coordinate axis (x, y, or z) [27]. FEA programs simulate fiber paths by assigning a material orientation to each element within the model. Models that neglect the effects of ply draping simulate fiber path in a contiguous manner, regardless of structural geometry. While this approach is accurate for simple flat geometries, it may not accurately account for fiber angle deviations that can occur as plies are draped over complex mold shapes [28].

**Figure 5:**
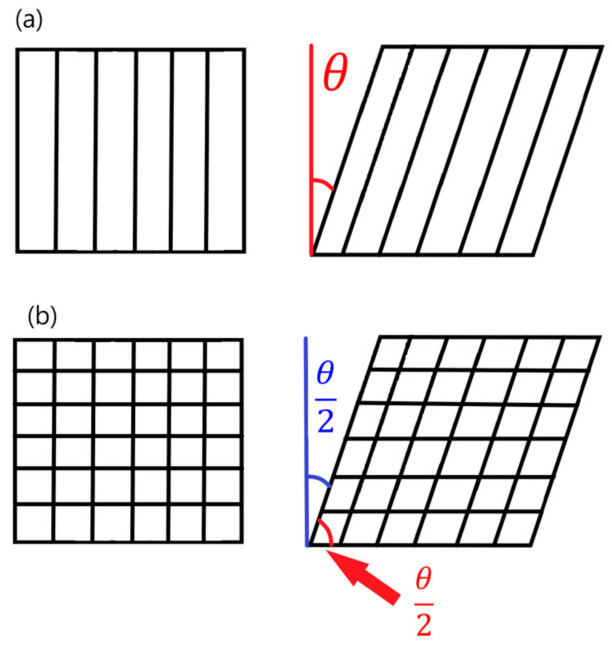
Local fiber orientations neglecting the effects of draping (**left**) and accounting for them (**right**) for unidirectional (**a**), and bi-directional plain weave (**b**) materials.

When flat ply shapes are draped over complex curved mold geometries, fiber angles can be locally skewed due to wrinkling of the ply. These fiber angle deviations are known as fabric shear [29]. During draping, the direction of fiber tows within the unidirectional tape can be locally altered. Similarly, the angle between the warp and weft in a bi-directional plain weave fabrics can be altered from the typical 90° to a geometry-specific value during draping. Figure 5a illustrates how unidirectional tape can be sheared and fiber angles can be altered by a value of *θ* [30]. For bi-directional fabrics, the fiber angles between the warp and weft are modified by a value of *θ*/2, as shown in Figure 5b. These local fabric distortions can create stress concentrations that can reduce structural performance, leading to increases in required weight and material costs to prevent failure [7]. Ply draping simulators can accurately predict where local fabric shear will occur based on structural geometry and re-assign material orientation within FEA model elements to represent the deviations in fiber angle.

Ply thickness variation is a related negative effect that can occur when draping plies over complex mold geometries. Wrinkling of a ply due to fabric shearing creates deviations in the ply thickness. Similar to fiber angle deviations, localized changes in ply thickness can create stress concentrations which reduce structural performance. During draping simulation, the ply thickness at each element location is calculated based on the degree of fabric shear, *θ*. The thickness of each element within a FEA model is given by Equation (1) [19]. Where t is the local ply thickness of an element following ply draping and t_0_ is the thickness of the ply when laid flat.

(1) $$t=t_{0}/\cos\left(\theta \right)$$

For large and complex structures, the effects of draping and fabric shear on structural performance can be significant [31]. To investigate these effects with respect to the anchor bracket, the first step was to develop two FEA models. In both models, plies were modeled with identical geometry to the overall part, forming an “integral” structure. Integral laminates were created for both models that included one ply for each material type and possible fiber angle orientation to assess the effects of ply draping on each of them. This study assumed all plies would be laid up in six typical orientations: 0°, 45°, −45°, and 90° for unidirectional materials and ±45° and 0°/90° for bi-directional weaves. With the four material types discussed in the previous section, this created laminates with 12 total plies.

**Figure 6:**
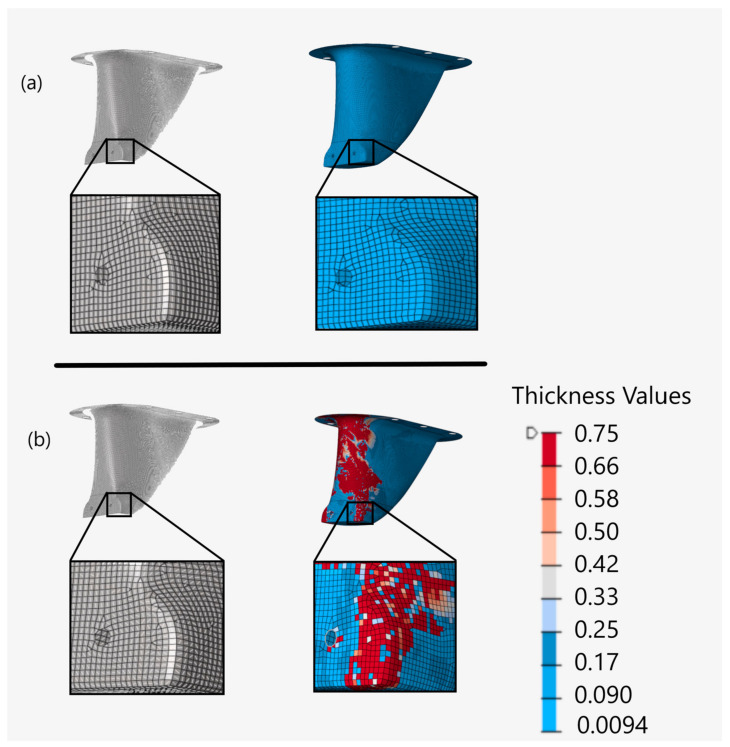
Example of unidirectional fiber angles left and ply thickness right without draping (**a**) and with draping (**b**) for integral models.

Next, fiber orientation within each element was defined. The first model neglected the effects of ply draping and instead aligned fibers with respect to the FEA coordinate system, consistent with most published composite research [27]. The second model, however, was generated through the ply draping simulator included in HyperWorks. Figure 6 provides an example of the difference in local fiber angles for the model that neglected draping and for the model that did account for it. In this example, the draping simulation predicted that a 0° unidirectional ply would have wrinkling in many areas, including near a mounting hole near the bottom of the structure. The model that was not draped, however, had contiguous fiber paths in the same region.

The same figure also demonstrates how fabric shear is directly related to ply thickness deviations due to wrinkling. The majority of the front of the structure on the draped model had increased thickness due to ply wrinkling, including near the mounting hole at the base of the structure. The model that neglected draping had no fabric shear, contiguous fiber paths, and uniform thickness throughout the structure matching the defined manufacturable ply thickness of 0.18 mm, provided in Table 1. Although slightly different, similar results were observed for both unidirectional plies oriented at 45°, −45° and 90°, as well as for the bi-directional fabrics. Thus, matching the ply geometries to the overall structural dimensions resulted in the issues observed in the draped integral model. These issues resulted in a significant difference between fiber orientations and performance when draping was, and was not, considered. The result is that weight savings and performance gains would be highly exaggerated without including draping in the analysis.

The wrinkling and fiber misorientation issues observed in the draped integral model can be considered a worst-case scenario. To improve the retention of fiber position and orientation, partitioning of plies to improve drapability would logically improve structural performance and reduce mass and costs of the structure compared to the draped integral model. To accomplish this, a process within the draping simulator known as kinematic draping was used to inform the partitioning of plies to improve drapability and create a third model. The kinematic draping process assesses the initial details of the integral plies and partitions them to minimize fabric shearing and ply wrinkling. The kinematic process indicated that partitioning the structure into two segments would produce optimal draping results.

**Figure 7:**
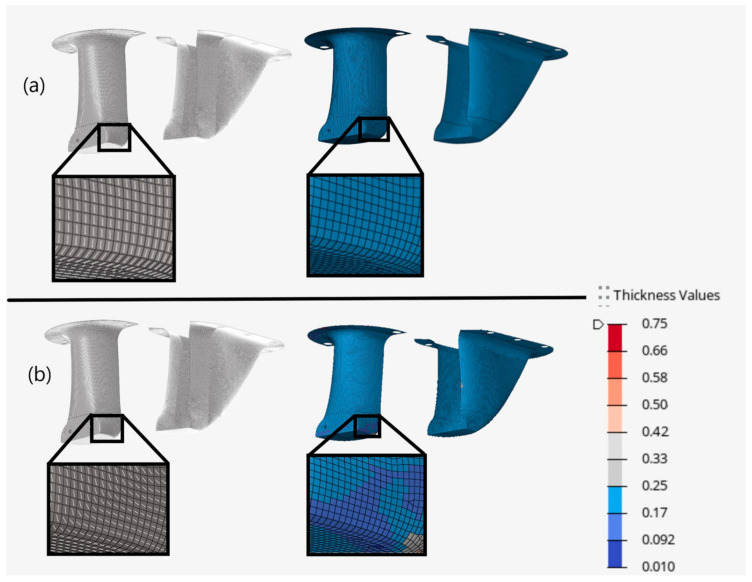
Example of unidirectional fiber angles left and ply thickness right without draping (**a**) and with draping (**b**) for partitioned models.

To assess the effects of ply draping on a partitioned structure, a fourth model was created with identical partitioning, but neglecting the fiber angle and thickness effects of draping on the plies. Figure 7 provides an example of the partitioned ply geometries, fabric shear, and thickness values for a 0° unidirectional ply. From this figure, it can be observed that partitioning the plies on the draped model improves fabric shearing and reduces ply thickness deviations when compared to the integral draped model presented earlier in Figure 6b. Figure 7a illustrates how neglecting ply draping will result in uniform ply thickness and no fabric shearing, consistent with the integral models.

**Figure 8:**
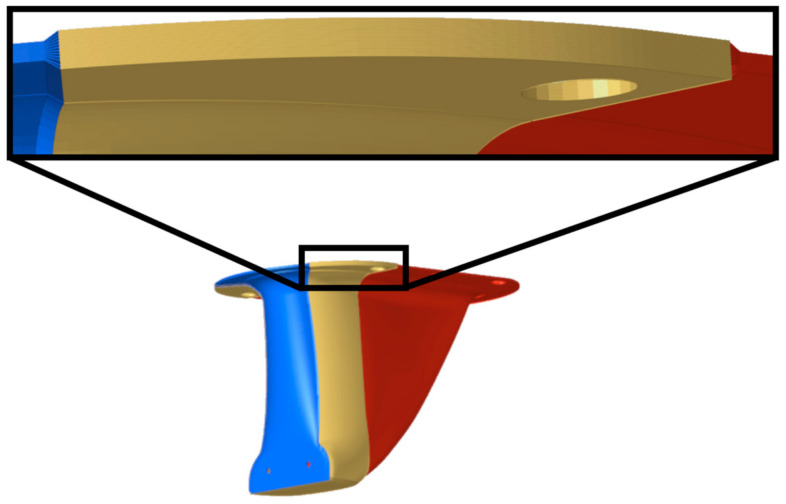
12-ply laminate regions shown in yellow and red, 24-ply laminate overlap region shown in blue, and detailed view of finger joints for the partitioned structures (top).

A feature not included in the kinematic draping analysis is the development of connections between the partitioned areas. This is, however, an important step in accurately modeling a composite structure, as joints can create stress concentration that reduces structural performance. Based on an analysis of the draping results, an overlapping region between the two partitioned sections was identified, where finger joints of a maximum length (126 mm wide at the narrowest point of the joint) could be formed without creating substantial fiber shear or wrinkling of the plies, as shown in Figure 8. The majority of the laminate for the partitioned structure was composed of a front and a back partitioned area, each with 12 plies. There were 24 plies in the developed finger joint region, stacked in alternating order with plies from the front and back partitioned sections.

### 2.4. Topology Optimization Formulation

With the four FEA models created, the next step was to formulate the topology optimization process. Industries that lack experience with the application of composites, such as the rail industry, have been researching and developing methodologies to assist in the design of composite structures [36]. Topology optimization (TO) has been the most widely researched optimization methodology applied within composites research, as it exploits one of the key manufacturing benefits of the material: ply tailoring [15]. During composite manufacturing, plies can be tailored into many complex and unique shapes and laid up within a laminate to reduce mass and material costs by maintaining thickness in areas prone to failure or that lack stiffness, while thinning the structure in areas with lower stress [37]. TO processes are governed by mathematical algorithms that are formulated to iteratively erode initial ply dimensions to mimic ply tailoring until a design solution is identified that meets established constraints. The methodology has been so widely applied that TO algorithms are now included in many FEA solvers, such as Altair’s OptiStruct, which was used in this research [38].

The objective function that governs the optimization algorithm can be customized within the FEA solver to meet a range of design goals. Recent research has begun formulating optimization functions to reduce the material costs or mass of composite structures but rarely have optimization functions been formulated to reduce both [39]. Furthermore, most of these studies have neglected the effect of material selection on the outcome of the optimization process [5]. Material selection is an important factor when attempting to optimize a composite structure, as composite material types range drastically in cost and material properties, as evidenced by the values listed in Table 1. In this study, a new optimization function is proposed to meet multiple objectives: to assist in material composition and reduce mass and cost. The objective function for this study is defined by Equation (2):

(2) $$f\left(x\right)=m_{1}c_{1}+m_{2}c_{2}+m_{3}c_{3}+m_{4}c_{4}$$

where m_1_, m_2_, m_3_ and m_4_ are the total masses of all plies within the structure of the four material types considered in this study, and c_1_, c_2_, c_3_ and c_4_ are the costs per kilogram for the materials, as listed in Table 1.

Optimization algorithms are also typically constrained to develop design solutions that meet other design and manufacturing requirements. Common constraints in structural design include composite failure criterion and compliance. For failure, the maximum stress failure criteria was used as a constraint for the optimization [40]. FEA software calculates failure criterion values for each element in the model. In this study, a failure criterion value of one was used as a constraint for the optimization process. This meant that no element within the structure could have a value greater than one (predicting failure) following the optimization process when the specified loads and boundary conditions are applied. As a highly conservative loading profile was used in this study, no safety factor was added to the failure criterion requirement.

The other common design constraint applied to optimization processes is compliance, C, which is the inverse of stiffness [41]. In this study, the overall structural compliance, 3.820 × 10^−9^ mm/N, of the existing steel structure was used as a constraint for the composite optimization.

From the manufacturing perspective, common constraints are ply thickness and discrete fiber angles [42]. Ply thickness is a common manufacturing optimization constraint, as it ensures that the developed design includes plies that have achievable thickness with the selected material and manufacturing process [43]. In this study, ply thickness was constrained to match the values listed in material testing reports and summarized in Table 1.

The other manufacturing constraint applied is the number of discrete fiber angle orientations the algorithm can select from. In this study, it was assumed that unidirectional materials could only be oriented at 0°, 45°, −45°, and 90° and bidirectional materials could only be oriented at 0°/90° and ±45°, which have been common constraints in other studies [44].

Once the objective function and constraints are defined, the full optimization algorithm can be defined. In this case study, the customized algorithm is defined by Equation (3).

(3) $$\begin{array}{c} \mathrm{minimize}\;f\left(x\right)\\ \mathrm{subject}\;\mathrm{to}\\ F\leq 1\\ C\leq 3.820\times 10^{-9}\;\mathrm{mm}/N\\ T_{\mathrm{UD}}=0.18\;\mathrm{mm}\\ T_{W}=0.19\;\mathrm{mm}\\ A_{\mathrm{UD}}=0{}^{\circ},\;45{}^{\circ},\;-45{}^{\circ},\;\mathrm{or}\;90{}^{\circ}\\ A_{W}=0{}^{\circ}/90{}^{\circ}\;\mathrm{or}\;\pm 45{}^{\circ} \end{array}$$

where f(x) is the objective function, defined in Equation (2), F and C are the failure and compliance constraints, T_UD_ and T_W_ are thickness constraints for unidirectional and woven materials, and A_UD_ and A_W_ are the fiber angle constraints for unidirectional and woven materials.

As previously explained, topology optimization erodes laminate geometries to identify minimal dimensions that meet the design goal and satisfy the constraints established. Therefore, before an optimization process can be initiated, the structure must at least meet all constraints established. The model that provided the most conservative starting ply dimensions was the draped integral structure, due to the predicted failure criteria for elements surrounding wrinkling and fabric shear. That structure required a maximum starting laminate thickness of 195 mm to meet the established constraints. The other three models were also set to match this thickness value for the integral draped structure, to normalize the process prior to starting the optimization. The 195 mm thickness of the laminate was equally divided by the number of plies in the model (either 12 or 24). This meant that, as a starting point, by mass, all models were equally composed of the four material types considered. This was done to ensure that material selection would not be biased towards any of the four types ahead of the optimization process.

Initially, the plies created by evenly dividing the laminate thickness were treated as candidate stacks of plies for the optimizer to select fiber angle and the final number of manufacturable plies. During optimization, the stacks that have been defined in the model were divided into thinner plies which satisfy the nominal manufacturable thickness listed in Table 1 and more accurately simulate the structural performance of the laminate. For the two models that considered the effects of draping, the ply thickness value was used as a reference, and local thickness within each ply was varied based on fabric shear, consistent with Equation (1). For the two models that neglected the effects of ply draping, all plies would be exactly the thickness defined in Table 1 following optimization, with no variation within the ply. The ply areas are also modified during the optimization process, and the order of plies within the laminate are reorganized based on geometry and fiber angle, to develop a design that meets the constraints and design goal with minimal cost and mass.

## 3. Results and Discussion

### 3.1. Structural Topology

**Figure 9:**
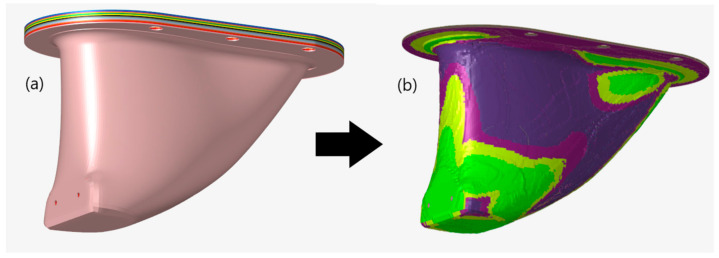
Example of ply geometry details before (**a**) and after (**b**) topology optimization for the integral model without draping.

The optimized versions of all four models achieved mass and cost savings through thickness reductions from the initial 195 mm thick laminate starting condition. As explained in the previous section, topology optimization achieved these reductions by eroding initial ply dimensions to meet the objectives of minimizing mass and material costs. Figure 9 provides an illustrated example of how the integral structure that neglected draping was optimized. In Figure 9a, prior to optimization, all plies had matching geometry to the overall structure. In Figure 9b, however, the ply geometries have been eroded to meet the optimization goals while still meeting the compliance and failure constraints.

**Figure 10:**
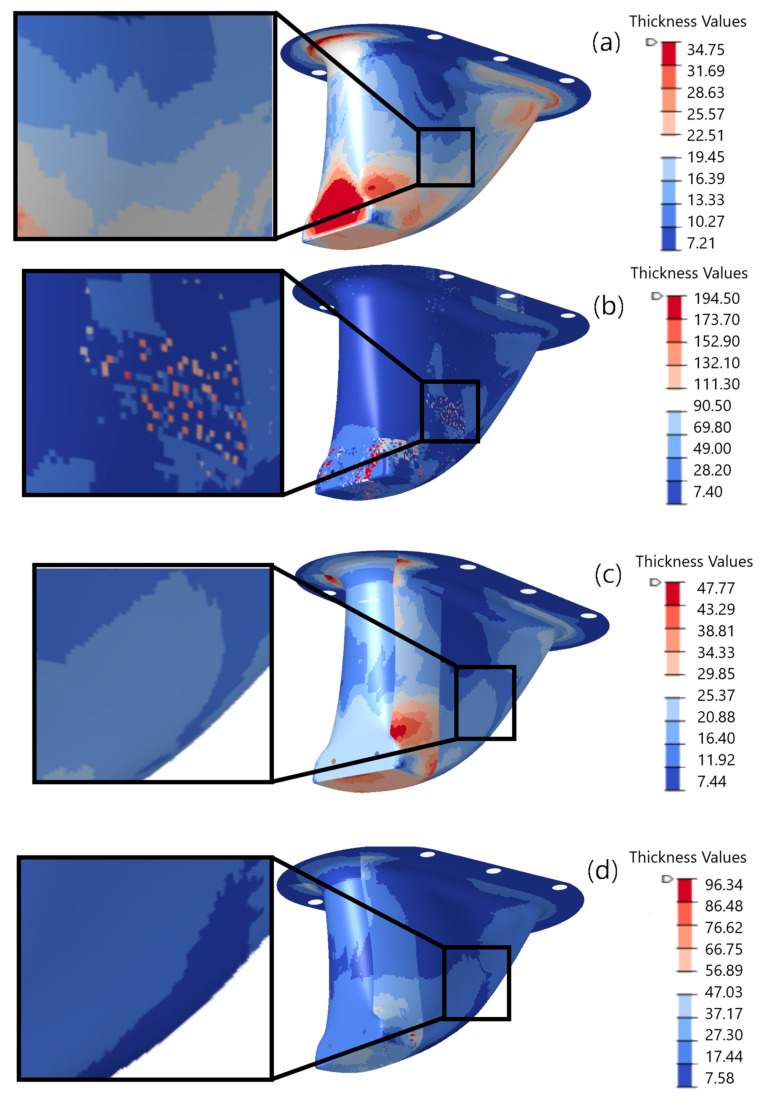
Laminate thickness contour plots for optimized models (**a**) integral without draping; (**b**) integral with draping; (**c**) partitioned without draping; (**d**) partitioned with draping.

For comparison, Figure 10 shows laminate thickness range plots of the four models following optimization. The figure also provides detailed views of small areas of the laminate to emphasize the thickness differences between the models. The detailed view shown in Figure 10a shows a maximum laminate thickness in that area of roughly 20 mm for the integral model without draping. Figure 10b shows the same detailed view area, but in the draped model, where a maximum laminate thickness of roughly 150 mm was present following optimization. Similarly, Figure 10c shows a maximum laminate thickness in a different detailed view area of roughly 20 mm for partitioned model without draping. Finally, Figure 10d shows a maximum laminate thickness of roughly 27 mm in the same detailed view area as Figure 10c for partitioned model with draping. The differences in laminate thickness in the detailed view areas between draped and not draped models can be directly attributed to ply wrinkling induced by fabric shear. Fabric shear reduces local structural performance, which dictates thicker sections in the draped models.

### 3.2. Optimization Constraints

The structures were constrained with respect to two parameters failure criterion and compliance. For the two models that did not include draping analysis, the optimization process maintained thicker sections in areas to support the failure criterion and compliance constraint. The introduction of fabric shear and wrinkling in the draped models, on the other hand, created additional structural area that had to maintain thickness to meet the constraints. This is because fabric shearing realigns fibers in non-optimal orientations and induces stress which increases failure criteria in these areas.

**Figure 11:**
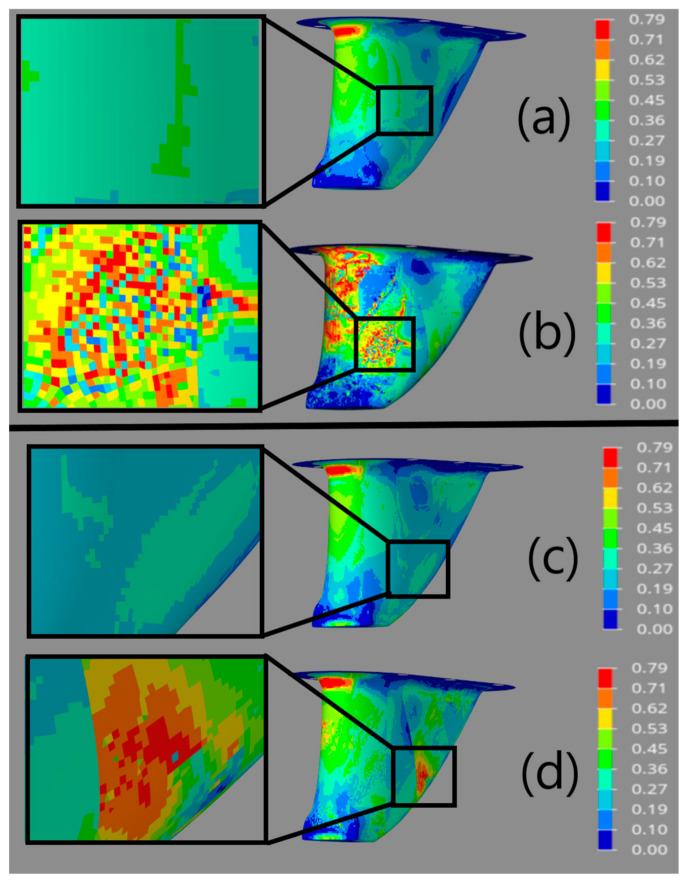
Maximum Stress failure criterion contour plots for optimized models (**a**) integral without draping; (**b**) integral with draping; (**c**) partitioned without draping; (**d**) partitioned with draping.

The optimization processes were constrained so that no element could have a failure criterion value of 1 or greater, based on the maximum stress failure theory. Figure 11 provides failure criteria plots for the four structures. The plots show the maximum failure criteria value predicted in any ply at all element locations in the structure following topology optimization. When comparing the results shown in Figure 11, it is evident that the structures that neglect ply draping fail to accurately predict failure in the structures. Figure 11 also provides detailed views of failure criteria values in the same areas that were highlighted in the thickness plots of Figure 10. When comparing the thickness ranges shown in Figure 10 to the failure criteria values shown in Figure 11, the topology optimization algorithm thickened the structural section in areas where failure was predicted. The models that neglected ply draping, however, were not thickened in the same areas, as elevated failure criteria values were not predicted. This demonstrates that the models that neglect draping would likely fail to perform as predicted following manufacturing, as they would not maintain thick enough local section to support the structural defects caused by ply draping.

It can also be noted from Figure 11 that the maximum failure criteria values on all four models were in the range of approximately 0.71–0.79, meaning that the failure criterion constraint was not the dominant constraint dictating section thickness in all areas of the structure. This means that structural thickness could have been reduced further in many areas and the constraint could have still been satisfied. Instead, the compliance constraint dictated the thickness requirements in many areas of the structure. Again, fabric shear causes the draped structures to maintain a thicker section to meet the required compliance, as fiber angle deviations reduce the stiffness due to the anisotropic nature of the materials and discontinuity of the fiber load paths. During optimization, the objective functions for all four models were reduced as far as possible to minimize costs and mass without violating the compliance constraint. As a result, all four structures had matching compliance meeting the optimization constraint value of 3.820 × 10^−9^ mm/N.

### 3.3. Mass Reduction, Material Selection and Costing

Prior to the topology optimization, the overall mass of each structure was evenly composed of the four candidate material types. The topology optimization algorithm that was formulated to assist with reducing the mass of the structures, selecting the optimal material for the designs and minimizing costs was successful in balancing all four designs between those competing objectives. With respect to mass reductions, as previously mentioned, all four structural masses were reduced through topology optimization. Due to the differences in fiber angles, ply thickness variability, and partitioning between the four models, the amount of mass savings achieved by each model varied. The two models that neglected the effects of ply draping achieved greater reductions in mass than the two models that did consider it. When comparing the integral models, the design which neglected draping achieved a 26% greater reduction in mass than the draped model. Similarly, when comparing the partitioned models, the design that neglected draping achieved a 6% greater reduction in mass than the draped model. This is an important outcome, as it demonstrates that partitioning is effective in allowing a draped model, which more accurately represents the real manufactured composite structure, to more closely meet the minimum mass predicted without any consideration of the manufacturing reality.

**Figure 12:**
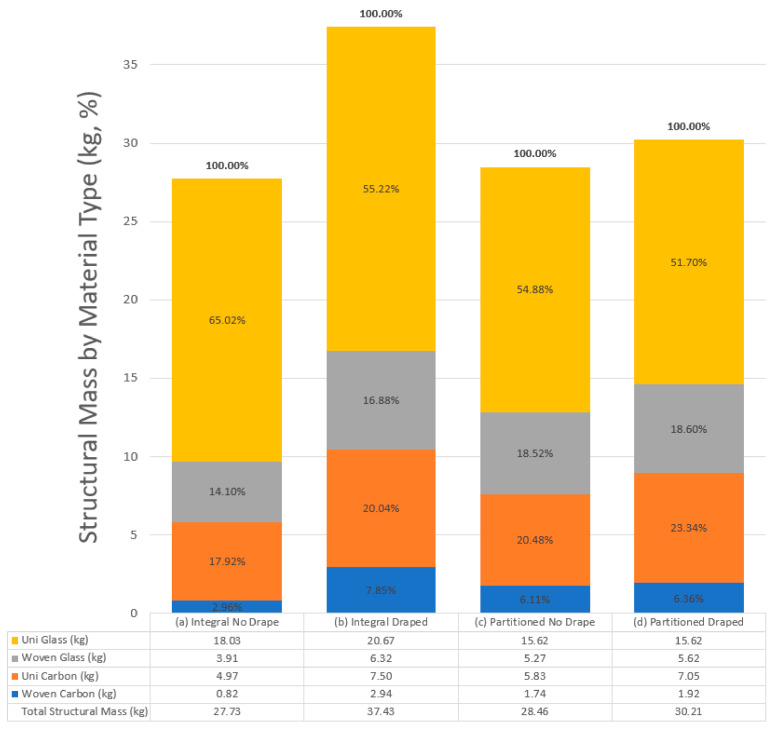
Comparison of optimized structural masses by material type for the four designs.

It is also logical that the models that did not consider ply draping could meet the compliance and failure constraints with less section due to uniform ply thicknesses and minimal fabric shear. This means that the mass saving results for these structures are unrealistic. For the draped structures, partitioning the laminate resulted in a 19% mass reduction compared to the integral design. The results for the draped structures are considered more realistic and achievable during manufacture. The table included in Figure 12 summarizes the total mass of each structure following optimization.

Figure 12 also summarizes the material selection process that was achieved during topology optimization. The stacked bar charts in the figure provide a breakdown by percentage of the total structural mass by material type following optimization. The table at the bottom of the figure also provides the mass composition in kilograms of each material following optimization. From this data, it can be noted that the algorithm favored unidirectional materials due to improved mechanical properties that assist in supporting the goal of minimizing mass, while meeting the design constraints of compliance and failure criteria. All four models had 51% or greater mass composition of unidirectional glass, and 17% or greater mass composition of unidirectional carbon.

**Figure 13:**
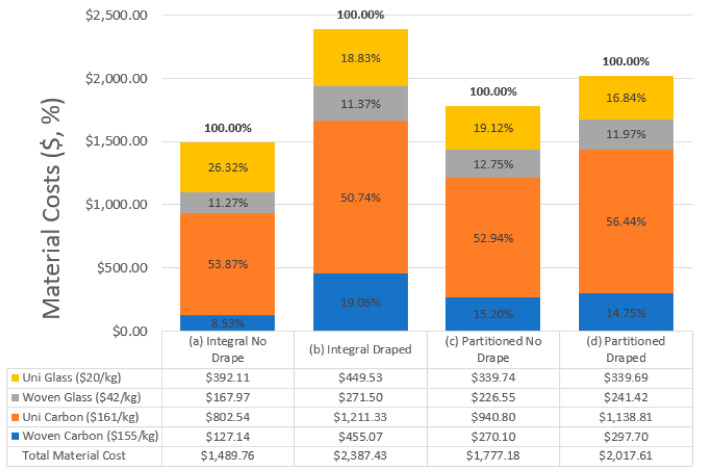
Comparison of structural cost by material type for the four designs.

With respect to cost minimization, the algorithm was also successful in reducing the costs of all four structures. Figure 13 summarizes the material costs for each structure following optimization and demonstrates how the algorithm balanced the solutions between the mechanical performance and the costs of each material type. For the glass fiber reinforced materials, unidirectional tape offers both improved mechanical performance and lower costs than the woven fabric, and therefore was prioritized in all four designs. For the carbon fiber reinforced materials, the unidirectional tape also offers improved mechanical performance over woven fabric. While the woven carbon fabric was cheaper, the relatively small difference in price ($6/kg) drove the algorithm to assess the material cost to mechanical property benefit of the materials and prioritize the unidirectional tape.

Like the results for structural mass, the structures that neglected ply draping predicted lower material costs than the draped models. When comparing the integral models, the design that neglected draping had a 38% lower material cost than the draped model. Similarly, when comparing the partitioned models, the design that neglected draping had a 12% lower material cost than the draped model. As noted for structural mass, the material costs for the draped partitioned structure were 15% lower when compared to the draped integral design. As discussed in relation to the mass results, the costs associated with the non-draped models would also be unachievable through actual manufacturing.

It should be noted that optimizing a fully glass fiber reinforced structure would likely provide greater cost savings, and a fully carbon fiber reinforced structure may provide greater mass reductions. By initiating the optimization process with the structures composed equally of the four materials, however, the algorithm was able to provide blended solutions that provided balance between the goals of reduced mass and lower material costs.

### 3.4. Kinematic Draping and Partitioning

With respect to the draped models, which are considered to be the most realistic and representative of manufacturable solutions, the kinematic draping process was effective in partitioning the structure. This is evident in that the partitioned structure provided additional mass and cost savings compared to the integral draped model. This is due to the heightened levels of fabric shearing and wrinkling in the integral model.

**Figure 14:**
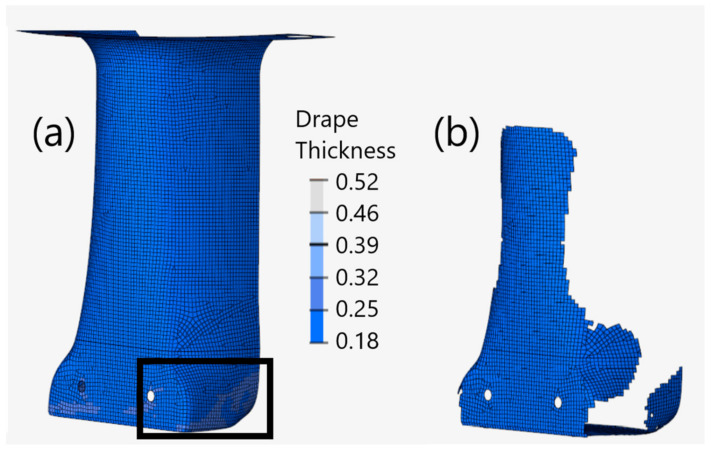
Example of thickness variation in one ply in the draped partitioned model before (**a**), and after (**b**), topology optimization.

The results of this study demonstrate the synergy developed when both topology optimization and draping simulations are combined and shows the benefits that can be achieved. Figure 14 provides an example of the benefits of combining the processes for the draped partitioned structure. Figure 14a shows an example ply following the kinematic draping process, but prior to topology optimization. While the kinematic draping process did greatly reduce fabric shear in the partitioned ply areas compared to the integral plies, some degree of thickness variations and wrinkling were still present in the structure. Prior to topology optimization, the example ply shown had thickness variations between 0.18 and 0.52 due to fabric wrinkling. The ply area in the lower right-hand corner, highlighted inside the black box in the figure, is where the majority of ply wrinkling occurred due to the complex curved geometry. Following optimization, however, the ply was tailored and the area where wrinkling occurred was eliminated, improving structural performance, as shown in Figure 14b. These types of complimentary improvements were made throughout the plies in the draped models. This demonstrates that combining topology optimization with draping analyses can assist designers in identifying ply geometries with improved drapability, guiding designs toward improvements in both structural performance and manufacturability.

### 3.5. Composite Joints

**Figure 15:**
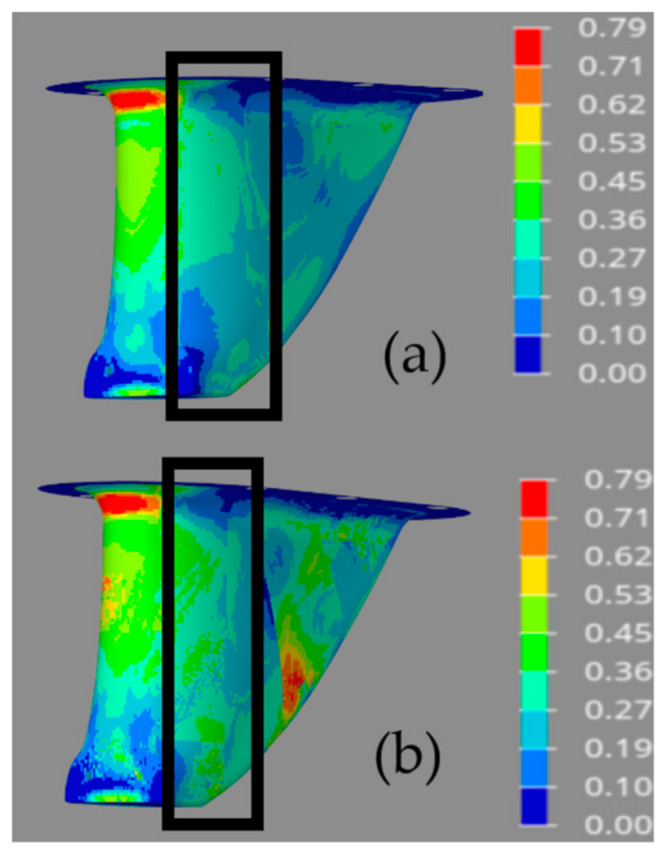
Maximum Stress failure criteria values in the finger joint area for the partitioned no drape (**a**) and partitioned draped models (**b**) following topology optimization.

Many studies have not accurately modeled connections between partitioned sections in composite structures [9]. This can lead to inaccurate projections on structural performance, mass and costs [45]. In this study, however, finger joints were accurately modeled for the partitioned structures. FEA predicted that the joints as modeled would perform well under the prescribed loading and boundary conditions. This is evident in Figure 15, which shows that, for the partitioned structures, maximum stress failure criteria in the joint area are in the range of 0.10–0.45, far below a range of a predicted failure.

**Figure 16:**
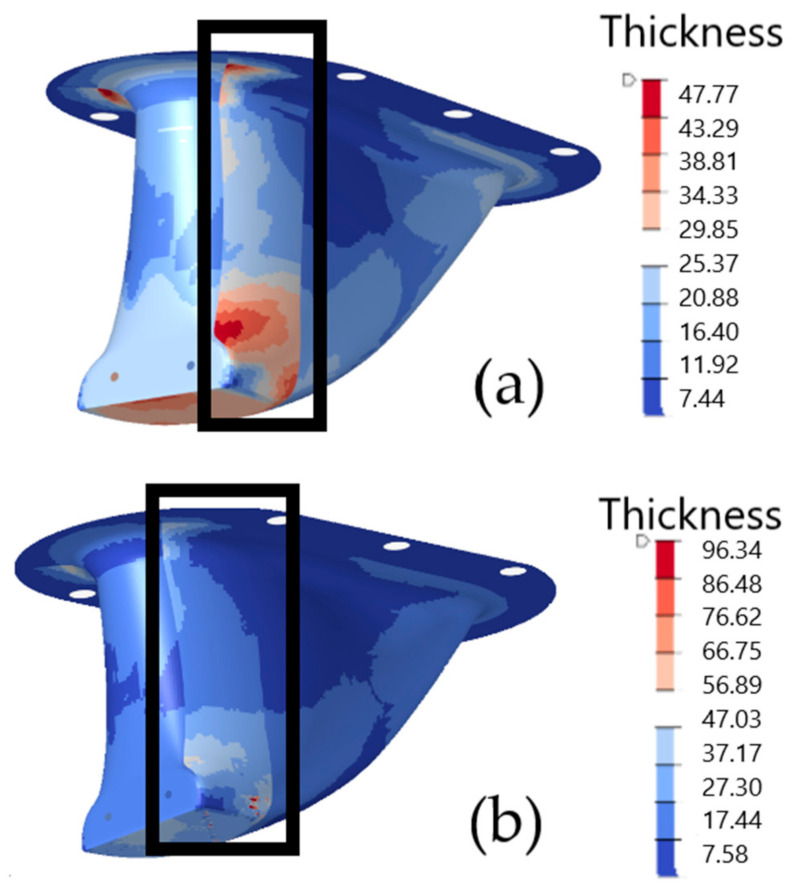
Thickness contour plots in the finger joint area for the partitioned no drape (**a**) and partitioned draped models (**b**) following topology optimization.

**Figure 17:**
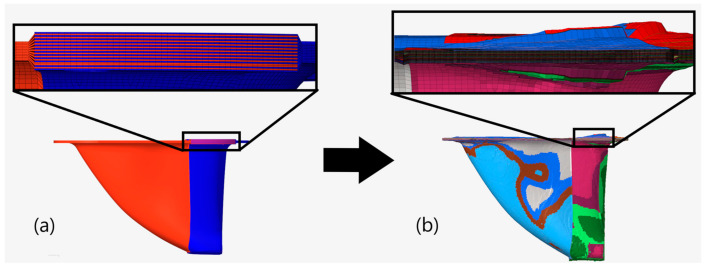
Finger joint ply geometry details before (**a**) and after (**b**) topology optimization.

The results of the topology optimization process also demonstrate benefits in joint design. Figure 16 illustrates that, for the partitioned structures, the joint topology was optimized. The joint thickness is tapered between the front and back partitioned ply areas. The topology optimization algorithm created these tapers to eliminate abrupt section changes between sections to reduce failure criteria values. This detail can be seen more clearly in Figure 17, which shows ply geometry details for the draped integral structure in the region of the finger joints, before and after optimization. This type of tapered transitioning in joints is common in composite design and is referred to as “ply drops”. In many published studies, ply drop designs have been developed through rules of thumb derived from experience, rather than in response to specific structural performance [46]. In this research, however, the ply drop details were generated automatically in response to structural performance through the optimization process. Therefore, this study demonstrates for the first time in published research the ability of topology optimization to inform composite joint and ply drop design.

### 3.6. Integrated Methodology

As evidenced in the results presented previously in this section, the integrated methodology of combining ply draping with topology optimization was successful in developing designs that balanced the key parameters of cost, manufacturing, mass, structural performance, and materials. When comparing between the two draped structures, the benefits of partitioning were evident, as the partitioned structure achieved a 19% lower mass and a 15% lower material cost than the integral design. Therefore, the draped partitioned structure developed through the integrated methodology represents the most blended, accurate, and manufacturable solution of the four structures considered.

In this research, the two designs that neglected the effects of ply draping were developed through topology optimization to represent current methodology presented in other research and used as a control for the experiment. In those studies, cost savings of over 40% and reductions in mass of 7% were reported [17,18]. As predicted, this research shows that neglecting the effects of draping, as was done in those past studies, however, can result in overly optimistic projections of structural performance, material costs, and mass reductions. The results of this research showed that neglecting ply draping will under-predict the mass of a structure by up to 26% and costs by 38%.

More recently, research has been published on the benefits of using ply draping simulations to inform partitioning of cost and weight efficient designs. Those studies reported 10% reduction in costs but a 13% increase in mass compared to the integral designs [21]. These mass increases were due to the increased material in connecting areas of partitioned structures. This study also did not incorporate topology optimization to minimize mass of the structure and the connecting joints.

When comparing the results achieved in this research to those published in past studies, the improvements offered by the integrated methodology are apparent. The draped and partitioned structure in this research offered 12% greater mass savings than the previously published partitioned structures where only topology optimization was conducted [17,18]. Furthermore, the draped and partitioned structure in this research offered a 5% greater cost savings and 32% greater reduction in mass over partitioned structures developed only through draping in past research [21]. Therefore, this integrated methodology demonstrates improvements over past published results in developing solutions which balance between cost, manufacturing, mass, structural performance, and materials.

The integrated methodology presented in this research was developed around commercially available FEA software packages. This was done to encourage rapid adoption of the methodology and to support industries that lack experience with composites in the design of structures. This methodology presents similar benefits to proprietary design tools and methodologies only available to designers in industries with extensive composite experience [12]. It is therefore envisioned that this methodology will assist in greater use of composites by other industries.

This study has developed new knowledge and innovation in several areas. First, topology optimization and kinematic ply draping methodologies were combined and integrated successfully. Second, a topology optimization algorithm was formulated to assist with material selection, and mass and cost minimization. Finally, the benefits of using topology optimization to assist with joint and ply drop design was demonstrated.

Despite the demonstrated benefits and new knowledge generated, the methodology could be improved in future research. First, the optimization function could be developed to provide additional material selection support to designers. For example, material types such as S-glass, high modulus carbon, and aramids could be considered. Furthermore, different feedstocks such as tows, twills and satin weaves could be included in the function. Additionally, this study only considered fiber reinforced composite material, but composite laminate designs with sandwich core materials such as honeycombs, foam and wood could also be added to the optimization function.

Combining more optimization parameters could also improve manufacturability. In this study, material properties and costs were based on hand layup and autoclave manufacturing processes. In future work, alternative manufacturing such as automated tape placement (ATP), resin transfer molding (RTM) or oven curing could be considered, which can result in different material costs and properties. The objective function could also be expanded to reduce costs associated with manufacturing such as tooling and labor. Additionally, the optimization process could be further constrained with respect to manufacturing. For example, in this study, the degree to which plies could be eroded and small and unique plies could be generated was not inhibited. While this does result in the maximum mass and material cost reductions possible, tailoring numerous plies into small complex shapes likely adds little to overall structural performance and would add considerable additional manufacturing labor effort. Therefore, a sensitivity analysis could be conducted to determine the cost/benefit related to the minimum size of unique ply geometries and an optimal degree of erosion to balance weight savings and cost-efficiency.

Finally, physical validation of the methodologies proposed in this study should be completed. While topology optimization and draping simulations have been validated in numerous past studies [9,28], the novel process of combining them proposed in this study may require adjustment based on physical experiments. This would likely be completed on a smaller and less complex structure.

## 4. Conclusions

Partitioning ply geometries within complex laminated structures through kinematic draping analyses can reduce manufacturing costs and improve manufacturability of the design. Additionally, topology optimization algorithms can be developed to further improve ply drapability and to simultaneously assist in materials selection. In this study, a new methodology was developed to combine topology optimization, material selection and ply draping simulations to partition complex composite structures with the goal of creating cost- and weight-efficient designs. This methodology was compared both against integral structures that were not partitioned and structures that did not consider the effects of ply draping. The results of this comparison showed that the partitioned structure had a 19% lower mass and 15% lower material costs than the integral design when considering the effects of ply draping. Further comparison to models that neglected the effects of ply draping demonstrated that doing so could result in designs that under-predict the mass of a structure by 26% and costs by 38%. The developed methodology demonstrates promise as a practice that can assist designers in the selection of composite materials and in developing cost- and weight-efficient structures.

## Figures and Tables

**Table 1:** Composite material properties.

Material Property	AS4 Unidirectional Carbon [32]	AS4 Plain Weave Carbon [33]	Unidirectional E-Glass [34]	Plain Weave E-Glass [35]
Compressive Modulus, E1_t_ (MPa)	162,095.74	66,051.77	38,610.64	24,545.34
Compressive Modulus, E2_c_ (MPa)	8963.18	66,603.36	8273.71	23,373.23
Poisson’s Ratio, ν	0.35	0.046	0.26	0.12
In-plane shear modulus, G_12_ (MPa)	4688.43	4964.22	4143.75	3723.17
Mass Density, *ρ* (kg/mm^3^)	1.5 × 10^−6^	1.5 × 10^−6^	1.8 × 10^−6^	1.8 × 10^−6^
Longitudinal tensile strength parallel to the fiber angle, X_t_ (MPa)	2558.51	768.90	1061.79	471.05
Compressive strength parallel to the fiber angle, X_c_ (MPa)	1731.48	5301.40	609.84	570.61
Transverse tensile strength normal to the fiber angle, Y_t_ (MPa)	64.05	36,551.88	30.99	424.57
Compressive strength normal to the fiber angle, Y_c_ (MPa)	285.72	781.24	117.97	475.46
In-plane shear strength, S (MPa)	91.56	55.91	71.98	67.65
Cost ($/kg) [25]	161	155	20	42
Manufacturable nominal ply thickness (mm)	0.18	0.19	0.18	0.19

## Data Availability

All data generated or analyzed during this study are included in this published article.
